# Mental Health During COVID-19: *Tam Giao* and Vietnam's Response

**DOI:** 10.3389/fpsyt.2020.589618

**Published:** 2021-01-08

**Authors:** Sean Small, Judite Blanc

**Affiliations:** ^1^Department of Applied Psychology, New York University Steinhardt School of Culture, Education, and Human Development, New York, NY, United States; ^2^Department of Population Health, Center for Healthful Behavior Change, New York University Grossman School of Medicine, New York, NY, United States

**Keywords:** Vietnam, cultural additivity, COVID-19, *tam giao*, community solidarity

## Abstract

COVID-19 is a novel infectious disease and global health crisis with major psychological implications. Of particular focus are the effects it will have on low- and middle-income countries (LMICs) as being under-resourced poses many challenges. Vietnam, a country with an estimated population of 97.33 million people, which until 30 July, 2020, had 459 confirmed COVID-19 cases with no fatalities but as of November 4th had 35 deaths, can be viewed as a model LMIC for other countries struggling with COVID-19. Employing key tactics such as transparency and effective communication, Vietnam was able to foster strong cooperation between government and citizens, contributing to its success during COVID-19. Moreover, Vietnamese resilience, attributable, in part, to “*tam giao,” a coexistence of* religious and philosophical Taoism, Buddhism, and Confucianism through cultural additivity, provides a unique mindset that other countries can learn from to adapt and even build psychological resilience against COVID-19 pandemic's psychological outcomes. We suggest countries prioritize transparency and communication to mitigate stigmatization and psychological distress that can result from quarantine and other interventions while promoting resources that provide accurate scientific information and psychological aid to citizens. We believe that *Tam giao* could be repurposed to relieve inevitable contradictions between values and lifestyles in the context of this devastating global health crisis.

## Introduction

SARS-CoV-2, or COVID-19, is a novel infectious disease of pressing global public health concern. Its high transmissibility with an estimated reproductive number (R_0_) of 2.5 and devastating outcomes in susceptible populations (i.e., the immunocompromised) have disrupted the healthcare systems, economies, and psychological well-being of hundreds of countries worldwide (1, 2). Moreover, as of 4 November 2020, there have been more than 44 million confirmed cases and more than 1 million confirmed deaths of COVID-19 (3). It is thus imperative for countries currently affected by COVID-19 or preparing to re-open to learn from successful nations to stymie the spread of the disease and concomitantly reduce subsequent physical and mental health consequences. Success is predicated on efforts to successfully develop and/or maintain vaccines and therapies quickly in the context of stressed public health systems as well as unequivocal, transparent communication promoting guidelines such as social-distancing. In order to prevent further harm, every sector must be involved. Of particular focus is the impact COVID-19 has and will have on low- to middle-income countries (LMICs). LMICs are under-resourced and particularly vulnerable to the consequences of outbreaks, so protecting them is vital to successfully overcome COVID-19. Sadly, some LMICs like Brazil, which as of 4 November 2020, has over 5.4 million confirmed cases and over 150,000 deaths from COVID-19, are suffering immensely (4). Brazil's outcome can be attributed to a fragmented response involving political denialism by President Bolsanaro and misinformation (5, 6). It is worth noting that judgements regarding Brazil's response and subsequent reviews are mixed, with some suggesting political blame to be biased and its response to be better than (or the same as) certain high-income countries (7, 8). Vietnam, on the other hand, appears to have controlled the virus and provides lessons that can be adapted by other countries to attenuate its global impact. The Vietnamese government's seemingly high efficacy in containing COVID-19 was likely facilitated by a complex culture that is collectivist, highly trusting of its government, open to adopting new ideas, and particularly prepared from past experiences with SARS- making it particularly distinct from individualist cultures of, for example, the United States (9).

We will proceed by describing in detail Vietnam's early response to COVID-19, followed by a description of cultural additivity and *tam giao*. Then, we will discuss mental health concerns surrounding COVID-19 and subsequent lifestyle changes as well as how *tam giao* may have played a role in psychological resiliency in Vietnam. It is worth noting that to our knowledge, there are no studies examining the exact impact of cultural additivity of *tam giao* on COVID-19 related resilience in Vietnam. A section disclosing limitations of our mini-review and a conclusion will round off our discussion.

## Vietnam's Resiliency to COVID-19

Being under-resourced and neighbors with China, where cases of COVID-19 initially emerged, Vietnam was in a precarious position of potentially having an explosion of COVID-19 cases (9). With an estimated population of 97.33 million people, which until 30 July, 2020, had 459 confirmed COVID-19 cases with no fatalities but as of November 4th had 35 deaths, it can be viewed as a model LMIC for other countries struggling with COVID-19 (10, 11). The country proved resilient through significant and early efforts to detect and contain the virus while employing clear and consistent messaging to citizens about risks and relevant steps to take (9, 12). Every aspect of Vietnam's response cannot be realistically adopted, however, as its one-party government and past experiences with SARS, for example, cannot be replicated swiftly (9). Detection of the virus involved early development of testing kits- the first being developed on 7 February 2020-and proliferation of testing capacity - with the number of sites nationwide increasing from two in January to 120 by May 2020 (9). A noteworthy achievement of Vietnam's containment response is the employment of an approach of identifying and quarantining potential cases based on epidemiological risk, rather than observability of symptoms (9, 13). This approach may have significantly contributed to Vietnam's resiliency in its understanding of the infectiousness of COVID-19 arising before symptom discernibility (9).

The high efficacy of the communication of public health information can be traced to strict enforcement against misinformation and apt use of existing technologies. Included in Vietnam's total government response was the mobilization of police forces to engender trust among communities, spread relevant information about the virus, and strictly enforce guidelines (14). Of importance is the community approval of suspending certain personal rights to combat the spread of the virus as Vietnam's strict enforcement (i.e., distributing fines to those spreading misinformation) (9, 14) may be incompatible in other countries such as the United States of America where people protested mandatory mask-wearing (14). Trust fostered between communities and police, or community policing, involved a relationship in which the latter would distribute essentials such as face masks and accommodate to locals required to quarantine, allowing residents to feel comfortable providing trustworthy contact-tracing information (14). Communication by the Vietnamese government was early, beginning on 9 January 2020- prior to any confirmed cases- and continued to involve consistent use of technologies such as widespread texts and messaging on Facebook (9). Successful apps such as “NCOVI,” a joint “neighborhood watch” project by telecom companies and the Ministry of Health, saw 1,040,000 downloads, facilitating efforts to contact trace and quarantine with real-time data (9, 15). Reacting to and managing a pandemic inevitably involves reliance on one's public health system. Vietnam's increasing yearly investments into its public health infrastructure and past experiences with SARS proved to prepare it for COVID-19 with its national emergency operations center and surveillance system (9). The center featured a strong system focused on epidemiological training and disease prevention (9). The surveillance system, though extremely helpful in its ability to gain data relevant to contact tracing in real-time and prevent severe widespread infections, may not be a feasible system to adopt by other countries as it raises concerns about privacy and personal rights (9). Part of Vietnam's early hospital management policies included attention to infection prevention among healthcare workers, which proved problematic during the 2003–2004 SARS outbreak with many deaths (9, 15). The result, after strengthened procedures and training, as of 30 June 2020, was just four healthcare workers having been infected (9). Although Vietnam's public healthcare system has not been strained or overwhelmed to the extent of other countries, its preparedness and subsequent success can be learned from. Countries should seriously consider investing in public health infrastructure to bolster preventative strategies (9).

## *Tam Giao* and the Vietnamese COVID-19 Response

*Tam giao*, or the Three Teachings, is essential to Vietnamese culture and especially folklore, which acts as a cultural transmitter influencing contemporary thought and behavior (16). The Three Teachings is a coexistence of fundamental understandings of Buddhism, Confucianism, and Taoism. The specifics of how each individual views, interprets, and practices each of the teachings is variable, so essential or “basic” concepts accepted by laypeople such as *karma* in Buddhism are acknowledged when discussing *tam giao* (17). Each philosophy can be described as culturally added in that they are incorporated regardless of whether they may or may not contradict each other (17). An example would be the coexistence of Confucianism and Taoism where the former revolves around harmonious relationships with other members of society and maintaining social structures and the latter entails leaving the constrictions of society for living freely in nature, *wuwei* (16, 17). The two philosophies seem to contradict, but are able to be followed simultaneously in that one can follow Confucianism while working and then find solace in nature outside of work, for example (17). Interestingly, the influence or presence of each philosophy is not equally distributed in *tam giao* (17). Rather, Vuong et al. in their analysis of differing levels of cultural additivity suggest a higher possibility of additivity of Confucianism and Taoism, and isolation of Buddhism, in folklore (17). Moreover, there could be a dominance of Confucianism over the other two religions, which when supplemented by positive outcomes being seen in folklore stories with Confucianism and the vice of lying, has implications for understanding contradictory behaviors in Vietnamese culture- contradictory in the sense that one acts in a way seemingly opposed to a followed philosophy (16, 17). Thus, *tam giao* can be defined as a distinctly Vietnamese culture allowing the presence of the three philosophies with Confucianism holding a stronger position over the other two. This inequality could be attributable to folktale collections having been written largely by Confucian scholars and Vietnamese Confucianism expanding and subsequently opposing Buddhism in the twelfth-thirteenth centuries and beyond (17). Taoism, with its lack of institutional form, may be able to coexist more easily with Confucianism in that it remains non-threatening politically (17).

*Tam giao* in folklore as a means to perpetuate and communicate societal norms and expectations is important for gaining insight into the complexity of human decision-making, especially discrepancies between folklore and real-world conduct (16). The aforementioned finding of Confucianism and lying resulting in positive outcomes for characters in stories suggests that Vietnamese culture may tolerate or even validate lying for the maintenance of social harmony (16). This complicates Vietnamese culture and behavior in the context of the coronavirus pandemic, where lying or spreading misinformation, for example, can have severely deleterious effects. Lying about the seriousness of COVID-19 to maintain calm, however myopic, could be supported by *tam giao*. The deployment of strict police enforcement of guidelines can be seen as having two functions with regards to *tam giao*. It may (1) combat the fallacy of denying the seriousness of the pandemic for common good and (2) coincide with maintaining social harmony in effectively combating COVID-19 by promoting solidarity. The efficacy and approval of community policing were bolstered by messaging by the government that framed combatting COVID-19 as fighting a common enemy (war rhetoric), which produced an inspirational community spirit to come together to adopt measures such as wearing a mask (9, 14). Reinforcing knowledge about the virus while promoting and aiding public health measures via mask distribution in the context of this community spirit may illustrate how *tam giao* was intricately involved in the success of community policing in Vietnam. *Tam giao* exemplifies the intricacy of culture, government response, human behavior broadly, but especially so in the midst of a global pandemic. Although direct appraisals of causation or generalizations of the interaction between *tam giao* and Vietnam's resiliency need further study, the Three Teachings may have facilitated the confluence of prepared, swift government response and community cooperation, trust, and harmony.

## Considerations for Mental Health

COVID-19, unlike the previous SARS disease, is markedly transmissible, necessitating swift and stricter interventions such as quarantining (1). Vietnam's broad response was successful in containing the spread of the virus, but poses considerations for subsequent precipitated mental health concerns specific to pandemic (responses) (18–24). Some studies of Vietnam have shown that social distancing and isolation measures can be associated with high rates of post-traumatic stress (20) and increased rates of depression/anxiety (24), especially those present in hospitals suspected of having COVID-19 symptoms (22). Quarantining, which Vietnam has been aggressively employing, can impose psychological strain related to stigma, financial constraint, and guilt (18, 19). Stigmatization, specifically health-related stigma that arises from the novelty and high transmissibility of a disease, carries mental health burdens both somatic and psychological (19, 21). In the context of Vietnam, front-line HCWs quarantined for more than 3 weeks at Bach Mai Hospital reported stigma that worsened with regards to domains of poor self-image and public attitudes (19). Strikingly, stigma centered around guilt toward family and friends was the most common (19). The global pandemic inevitably presents and exacerbates mental health challenges, Vietnam as no exception. Although mental health programs are needed in Vietnam to address the psychological impacts of COVID-19, we will consider some of the (possible) protective factors that can be learned from their response.

Vietnam's swift unified response in the context of a resilient culture of additivity and *tam giao*, specifically, may have provided protective psychological factors such as health literacy, community solidarity and positive attitudes toward (the government's efficacy in) preventing further spread of COVID-19 (10, 22, 25–27). Interventions aimed at reducing psychological burden during COVID-19 depend in part on the repetitive dissemination of relevant information, total coordination of multiple health sectors, and promotion of altruistic behavior (18, 28). Having sufficient knowledge about the virus is necessary for epidemiological purposes and may correlate with increases in positive attitudes toward the virus (26). Moreover, this can be translated to health literacy, which can lower the likelihood of depression and increase measurements of quality of life in outpatients suspected of having COVID-19 symptoms (22). The Vietnamese government's rigorous campaign to combat misinformation alongside consistent equivocal messages about the virus may have protected against the effects of quarantine, facilitating optimistic attitudes and trust in the government, reducing catastrophic appraisals of health (18, 26). Communication by the government was laden with surprisingly effective war rhetoric (9, 25). Avoiding scapegoating or individualist solutions that can result from using war as a metaphor, Vietnam fostered community solidarity (25, 27). This ethical collective patriotism acts makes sense in maintaining the social harmony predominant in *tam giao*. Its effects to influence behavior and promote resilience can be seen with three-fourths of a Vietnamese sample implementing all six preventative measures and philanthropists supporting vulnerable groups with “rice ATMs” (23). Social solidarity can also be framed to buffer the mental health effects of viewing social distancing as social isolation, especially for vulnerable populations (25). Solidarity on its own cannot account for this, however. The success of the community solidarity seen in Vietnam can be seen as having included the ethical values of care and responsibility (25). These latter values are consistent with *tam giao*. Care, especially toward elder populations, who in this case are particularly vulnerable to COVID-19, is inextricable from Confucianism. Responsibility can be framed as fulfilling Confucian and/or Buddhist philosophies in that one has a duty to maintain the well-being of his community and perform good deeds such as social distancing as a form of good *karma*, for example. It is worth noting that acting responsibly may follow the former perspective as Confucianism “dominates” over Buddhism in *tam giao* (17). Additionally, Taoist philosophies of flowing in nature may function to uphold social distancing as behaviors to be alone concurrently reduces the spread of COVID-19. Community solidarity against COVID-19 in Vietnam, incorporating ethical values and practices that correspond with *tam giao*, may have functioned as community resilience to the psychological impact of the pandemic (29). Community policing facilitating greater tracking efforts by citizens may be an example of collaborative efforts contributing to Vietnamese collective solidarity (14). The multisectoral mobilization of the Vietnamese government, effectively disseminating quality information (and stopping widespread misinformation) about the pandemic and fostering collective wisdom/knowledge about practices and optimistic attitudes toward government capability, fit into a framework of collaborative positive psychology (9, 26, 30). Collaborative positive psychology is a model that highlights how negative emotions in a time of crisis can motivate positive collaborative transformation in which problem-focused and method-driven responses supporting sustainable well-being are predicated on key principles of collective solidarity, empowerment, intelligence, and teamwork (30). Sustainable well-being necessarily involves psychological health. *Tam giao* may have acted as a cultural catalyst undergirding community solidarity in Vietnam in response to the pandemic. Its promotion of collectivist ethics in the backdrop of altruistic acts and swift public health interventions may shape salutogenesis and ultimately have acted as psychological resilience (23, 30). The adaptability of Vietnam may be attributable, in part, to a turbulent cultural history of additivity in the face of many abrupt ideological changes (10). *Tam giao*, thus, may be inseparable from Vietnamese resilience and solidarity.

## Limitations

We acknowledge that there are many limitations to this mini-review. This review is not systematic and does not have concrete data measuring changes in the mental health of the Vietnamese throughout the global pandemic. Moreover, we were unable to find literature explicitly connecting *tam giao* and its impacts on resilience or psychological health in Vietnam. In light of this, however, we believe this can motivate further research into cultural additivity and *tam giao*, as well as their place in community and individual mental health and resilience.

## Conclusions

Vietnam has been effective in containing the spread of COVID-19 early, this being facilitated by swift government response and community resilience facilitated by its collectivist and additive culture. A unified response involving multi-sectoral coordination including community policing and acquisition of real-time data allowed Vietnam to control the spread of COVID-19 (9, 14). *Tam giao* and its ethical values and philosophies may have aided the efficacy of resulting health literacy, community solidarity, and positive outlook in relieving some of the psychological impacts of the pandemic. Information is extremely important for resilience against the psychological consequences of the pandemic (6, 18, 20). The psychological consequences of COVID-19 are beginning to become more clear with post-traumatic stress, depression, anxiety, and other symptoms associated with quarantine and social-distancing being observed (15, 18, 20, 21). Vietnam was (and still is) successful in responding to COVID-19, but, inevitably, did not completely avoid these psychological consequences (15, 20, 21). However, the phenomena of cultural additivity, involving the adoption of seemingly contradictory values, and *tam giao*, culminating in a collectivist manifestation of resilience, solidarity, and additivity and its role in response to COVID-19, may offer inspiration for future measures by other LMICs.

## Author Contributions

SS was tasked with research and writing of the abstract and manuscript. JB was tasked with overseeing and assisting the work of SS, providing edits and feedback. Both authors contributed to the article and approved the submitted version.

## Conflict of Interest

The authors declare that the research was conducted in the absence of any commercial or financial relationships that could be construed as a potential conflict of interest.
